# Supplementary material for: Morphological, Anatomical, and Phytochemical Studies of Carlina acaulis L. Cypsela
Source: Int J Mol Sci. 2020 Dec 3;21(23):9230. doi: 10.3390/ijms21239230 (PMC7730301; doi:10.3390/ijms21239230)
Supplement: Supplementary file 1 [file ijms-21-09230-s001.pdf]

SUPPLEMENTARY MATERIALS

Morphological, anatomical, and phytochemical studies of *Carlina acaulis* L. cypsela

Maciej Strzemski [1*], Bartosz J. Płachno [2*], Barbara Mazurek [3], Weronika Kozłowska [4], Ireneusz Sowa [1], Krzysztof Lustofin [2], Daniel Załuski [5], Łukasz Rydzik [6], Dariusz Szczepanek [7], Jan Sawicki [1], Magdalena Wójciak [1]

[1] Department of Analytical Chemistry, Medical University of Lublin, 20-093 Lublin, Poland; kosiorma@wp.pl (M.W.); i.sowa@umlub.pl (I.S.); 91chem91@gmail.com (J.S.)

[2] Department of Plant Cytology and Embryology, Institute of Botany, Faculty of Biology, Jagiellonian University in Kraków, 30-387 Cracow, Poland; bartosz.plachno@uj.edu.pl (B.J.P); krzysztof.lustofin@doctoral.uj.edu.pl (K.L.)

[3] Analytical Department, New Chemical Syntheses Institute, 24-110 Puławy, Poland; barbara.mazurek@ins.lukasiewicz.gov.pl (B.M.)

[4] Department of Pharmaceutical Biology, Wroclaw Medical University, 50-556, Wroclaw, Poland; weronika.kozlowska@umed.wroc.pl (W.K)

[5] Department of Pharmaceutical Botany and Pharmacognosy, Ludwik Rydygier Collegium Medicum, Nicolaus Copernicus University, 85-094 Bydgoszcz, Poland; daniel_zaluski@onet.eu (D.Z.)

[6] Faculty of Physical Education and Sport, Institute of Sport, University of Physical Education in Krakow, 31-541 Kraków, Poland; lukasz.gne@op.pl (Ł.R.)

[7] Chair and Department of Neurosurgery and Paediatric Neurosurgery, Medical University of Lublin, 20-090 Lublin, Poland; dariusz.szczepanek@umlub.pl (D.SZ.)

* Correspondence:
maciej.strzemski@poczta.onet.pl; Phone: +48-81-448-7180 (M.S.);
bartosz.plachno@uj.edu.pl; Phone: +48-12-664-60-39 (B.J.P.)

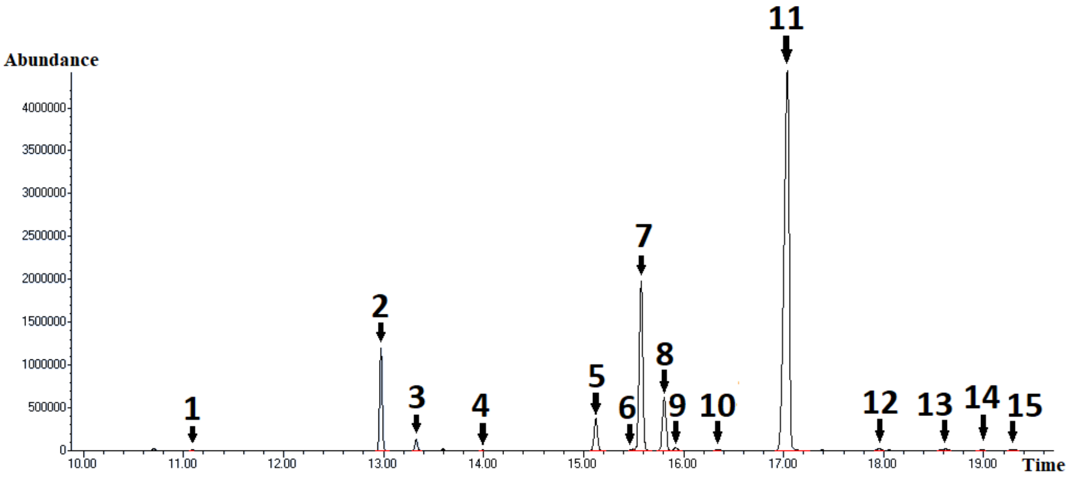

Figure S1. Example of a GC-MS chromatogram of *Carlina acaulis* cypsela oil. 1 – Myristic acid, 2 – Palmitic acid, 3 – cis-5-hexadecenoic acid ?, 4 – Margaric acid, 5 – Stearic acid, 6 – Unidentified compound, 7 – 5-Octadecenoic acid, 8 – Oleic acid, 9 – 8-Octadecenoic acid or 11- Octadecenoic acid, 10 – 9,12-Octadecadienoic acid, 11 – Linoleic acid, 12 – Arachidic acid, 13 – alpha-Linolenic acid, 14 – 11,14,17-Eicosadienoic acid or 9,12-Octadecadienoic acid, 15 – Mangiferic acid. HP-88 Agilent capillary column (60 m × 0.25 mm; 0.20 μm film thickness). The oven temperature was programmed from 110 °C to 190 °C with 8 °C/min, hold for 2 min at 110 °C and 13 min at 190 °C. The temperature of the injector was 250 °C. Helium was used as a carrier gas at a flow rate of 1.2 mL/min A quadrupole mass spectrometer with electron ionization (EI) at 70 eV and with a full scan type acquisition mode (50 m/z to 500 m/z) was used as a detector connected with the GC. The temperature of the MS source and the MS quadrupole was set to 230 °C and 150 °C, respectively.

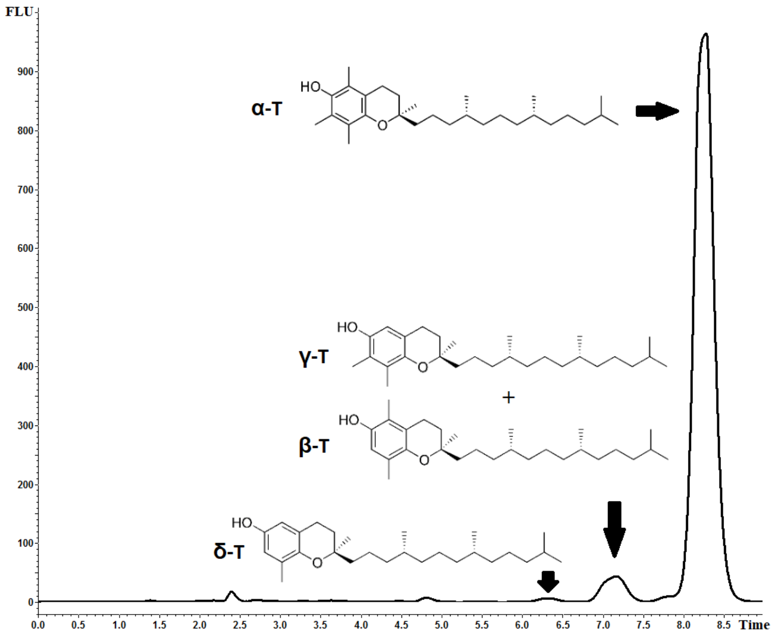

Figure S2. Example of an HPLC-FLD chromatogram of *Carlina acaulis* cypsela oil. The analysis was performed on an RP18e LiChrospher 100 column (25 cm x 4.0 mm i.d., 5 μm particle size). The column temperature was set to 30 °C. Acetonitrile and methanol (5:95 v/v) at a flow rate of 1.2 mL/min were used as eluent. λex=296 nm, λem=330 nm. α-T – alpha-Tocopherol, β-T – beta-Tocopherol, γ-T – gamma-Tocopherol, δ-T – delta-Tocopherol.

Table S1. Mass spectra for standards (based on the NIST database) and investigated fatty acids

Spectra of standards substances	Spectra of investigated compounds
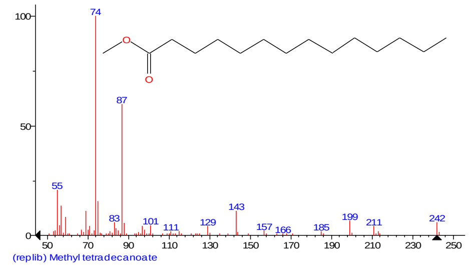 Myristic acid methyl ester	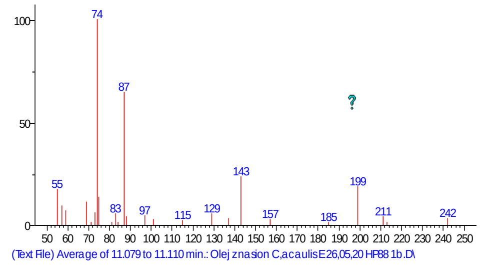
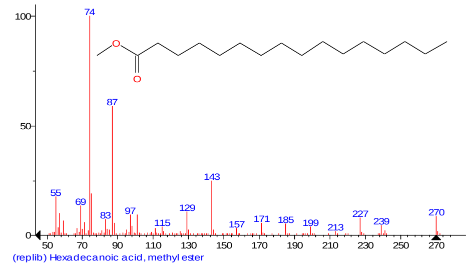 Palmitic acid methyl ester	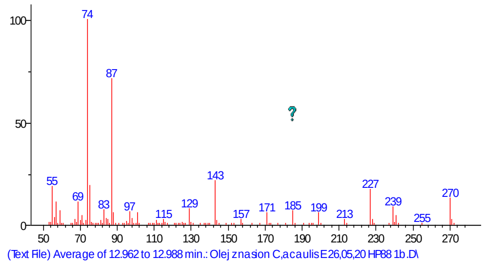
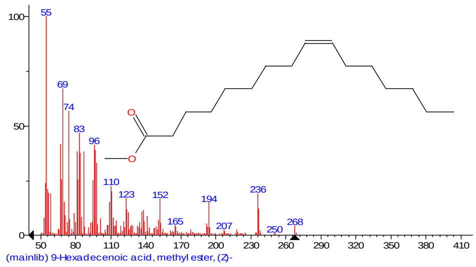 Palmitoleic acid methyl ester	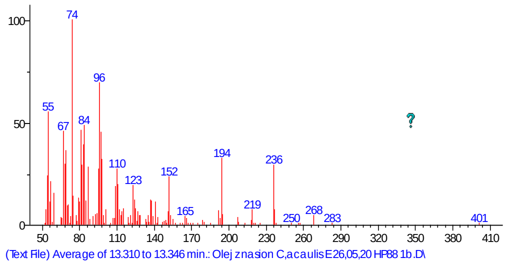 cis-5-hexadecenoic acid ?
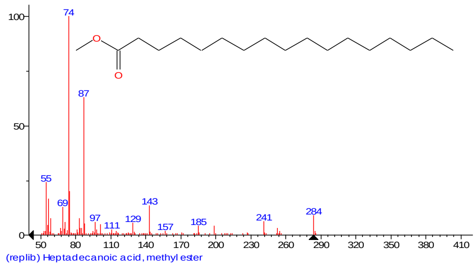 Margaric acid methyl ester	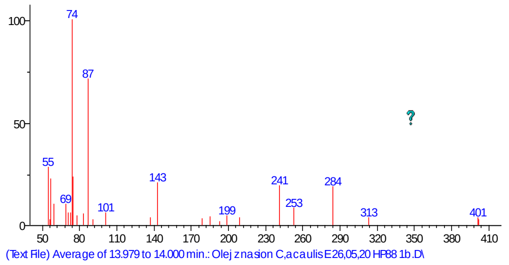
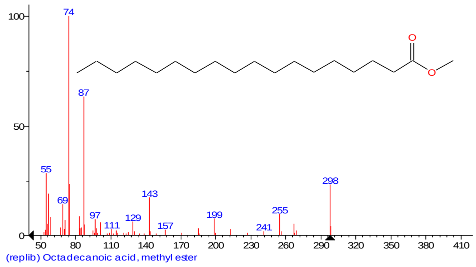 Stearic acid methyl ester	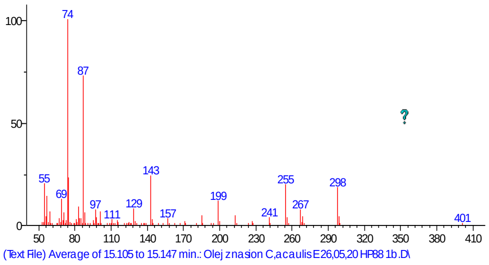
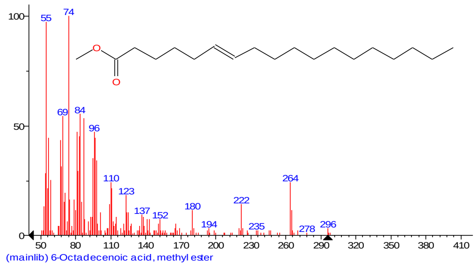 6-Octadecenoic acid methyl ester	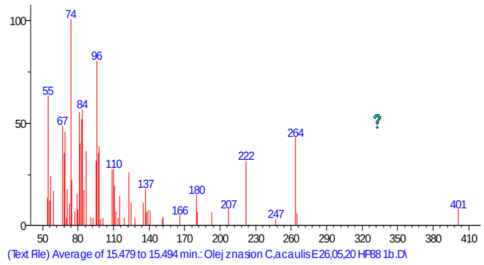

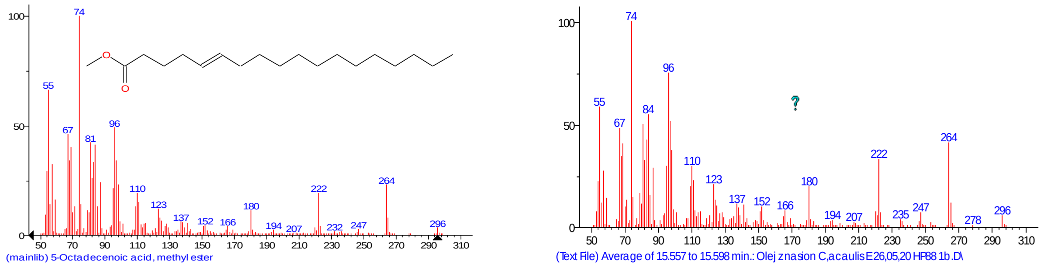

5-Octadecenoic acid methyl ester

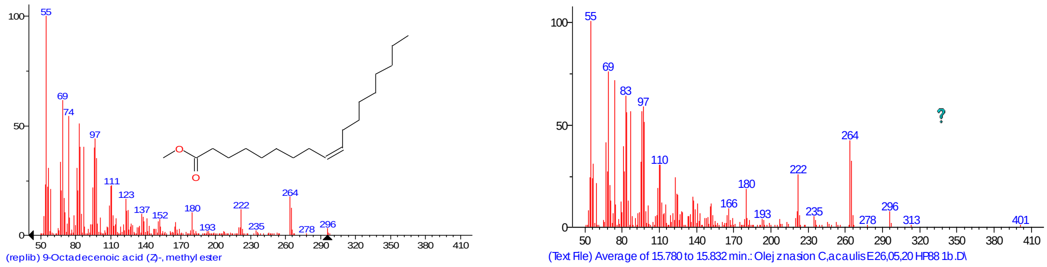

Oleic acid methyl ester

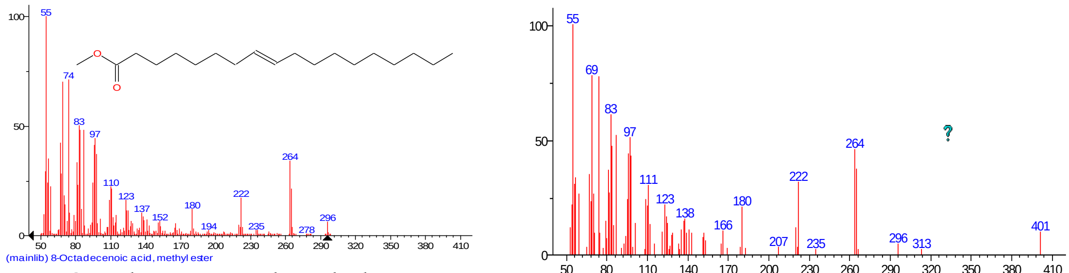

8-Octadecenoic acid methyl ester

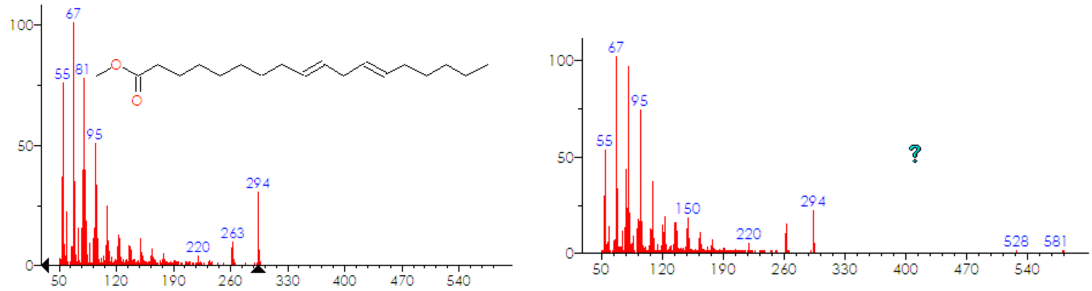

9,12-Octadecadienoic acid methyl ester

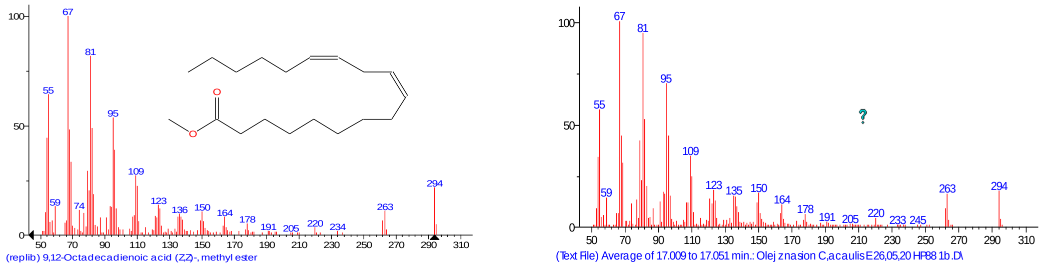

Linoleic acid methyl ester

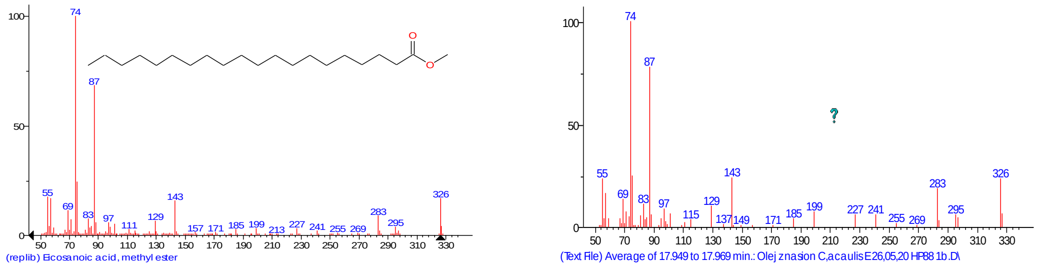

Arachidic acid methyl ester

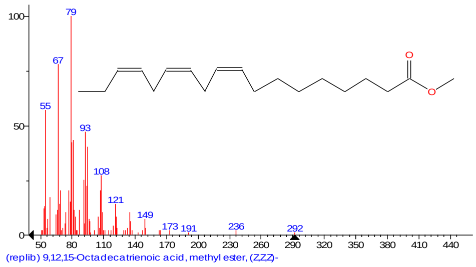

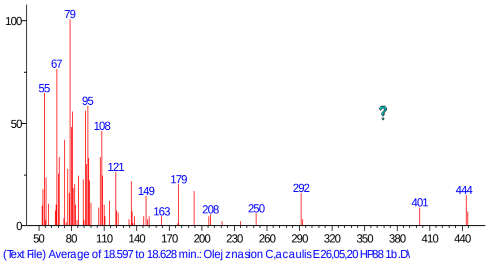

alpha-Linolenic acid methyl ester

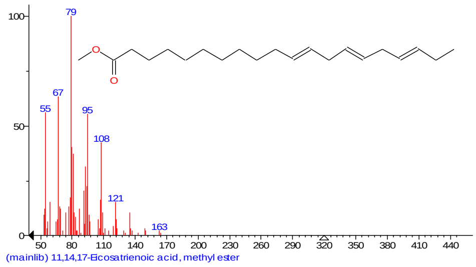

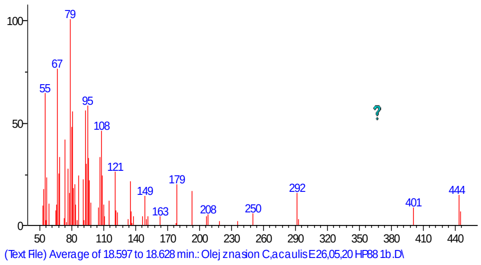

11,14,17-Eicosatrienoic acid methyl ester

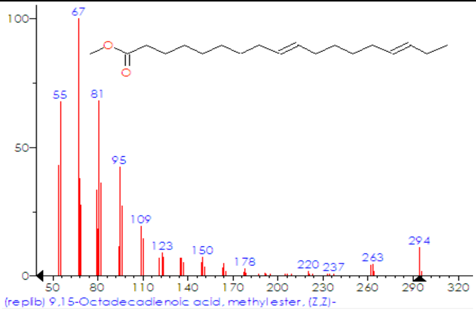

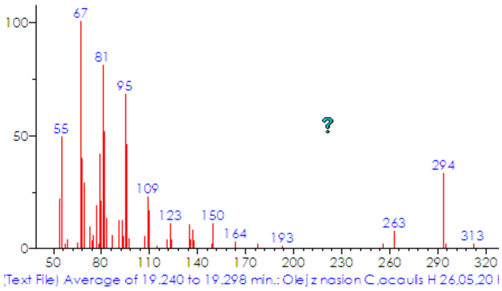

Mangiferic acid methyl ester

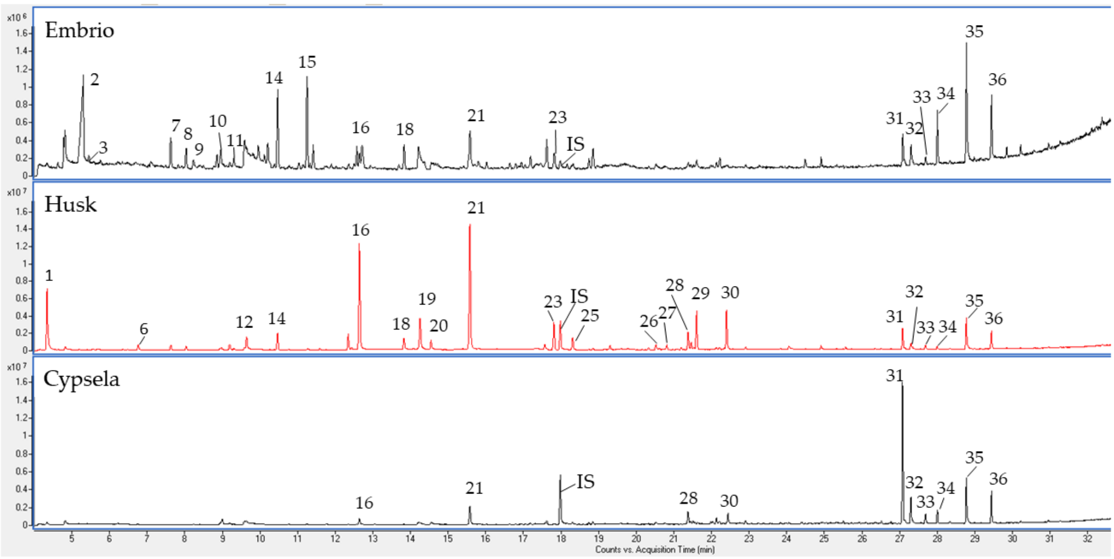

Figure S3. Example of a GC-MS chromatogram of volatile compounds in *Carlina acaulis* cypselae. 1 – hexanal, 2- 3-Methylbutanoic acid, 3 - 2-Methylbutanoic acid, 4 – 2-Hexenal, 5 - Pentanoic acid, 6 – Heptanal, 7 – α-Thujene, 8 – Camphene, 9 – γ-Valerolactone, 10 – Hexanoic acid, 11 – 2-Pentylofurane, 12 – Octanal, 13 – Limonene, 14 – Eucalyptol, 15 – 4,5-Dimethylnonane, 16 – Nonanal, 17 – 2-Ethylhexanoic, 18 – Camphor, 19 – 2-Nonenal, 20 – Octanoic acid, 21 – Decanal, 22 – Nonanoic acid, 23 – Bornyl acetate, IS – 2-Undecanone (internal standard), 25 – Undecanal, 26 – Tertadecane, 27 – Dodecanal, 28 – trans-Geranylactone, 29 – Alloaromadendrene, 30 – Tridecanal, 31 – Isopropyl myristate, 32 – Farnesyl acetaldehyde, 33 – Phtalic acid, hept-4-yl isobutyl ester, 34 – Nonandecane, 35 – 2-Ethylhexyl octadecyl carbonate, 36 – Isopropyl palmitate, 37 – Heneicosane, 38 – 2-Ethylhexyl 4-methoxycinnamate. The analysis was performed using Agilent 7890B GC coupled with the 7000GC/TQ system (Agilent Technologies, Paolo Alto, CA). Separation was carried out on an HP-5 MS column; 30m × 0.25 mm × 0.25 µm (J&W, Agilent Technologies, Palo Alto, CA) at a constant helium flow of 1 mL/min. The injector temperature was set at 250°C and the sample was applied in a split mode (20:1). The temperature program was 50°C for 1 min, followed by 4°C/min to 130°C, 10°C/min to 280°C, and held isothermal for 2 min. The MS source was set at 230°C, the transfer line was 320°C, and the quadrupole temperature was 150°C. The electron ionization energy was set at 70 eV, scan range, m/z 30-400.

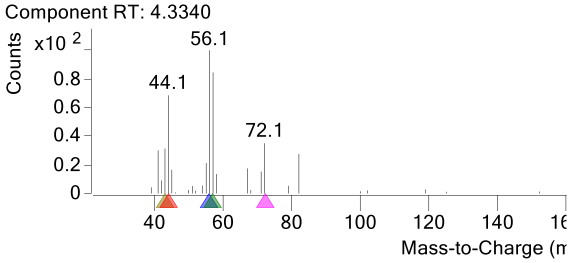

1. Hexanal

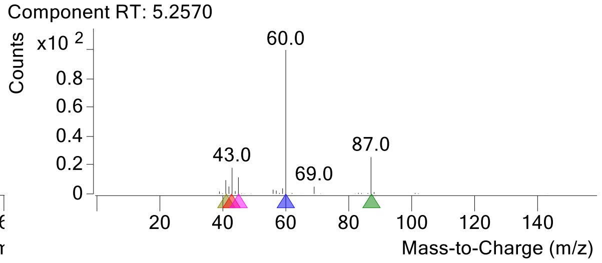

2. 3-Methylbutanoic acid

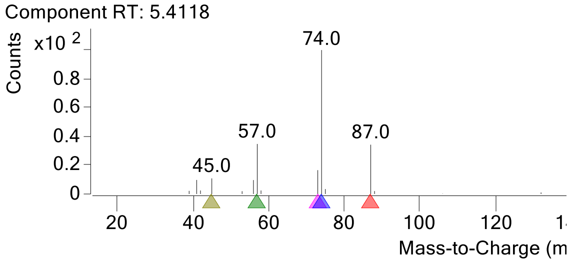

3. 2-Methylbutanoic acid

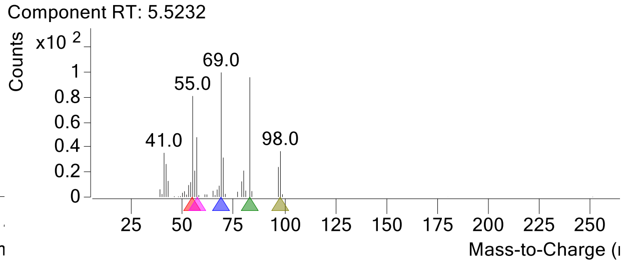

4. 2-Hexenal

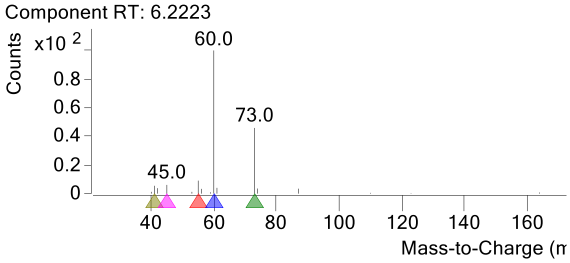

5. Pentanoic acid

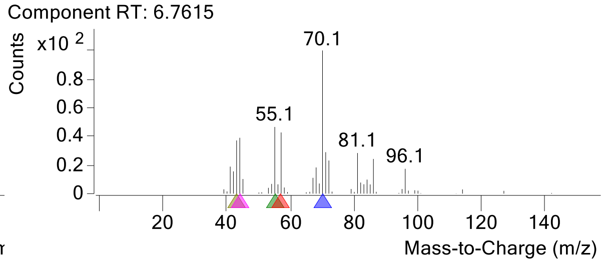

6. Heptanal

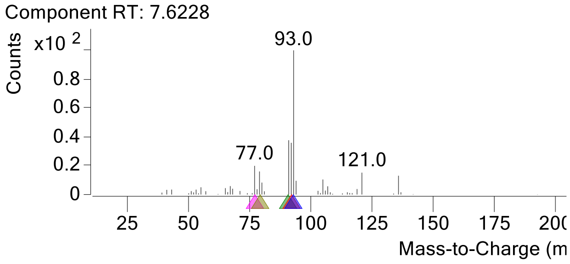

7. α-Thujene

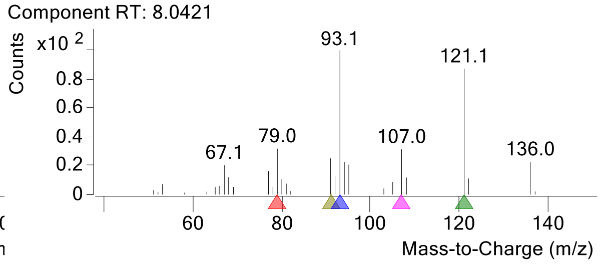

8. Camphene

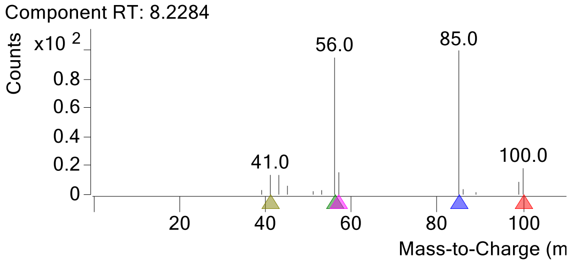

9. γ-Valerolactone

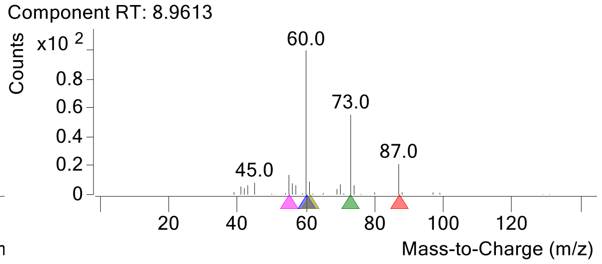

10. Hexanoic acid

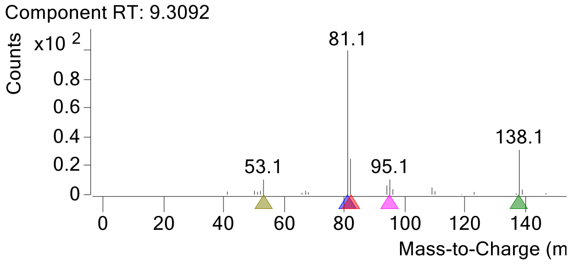

11. 2-Pentylfurane

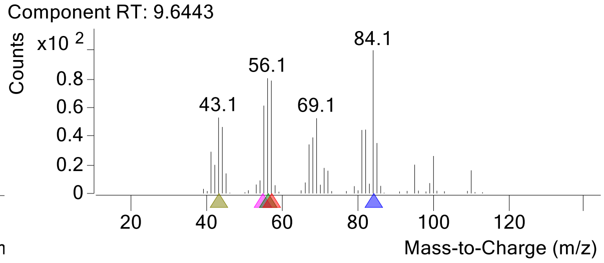

12. Octanal

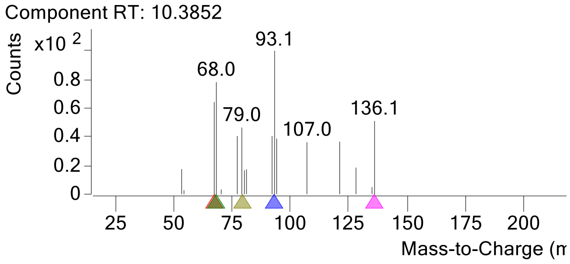

13. Limonene

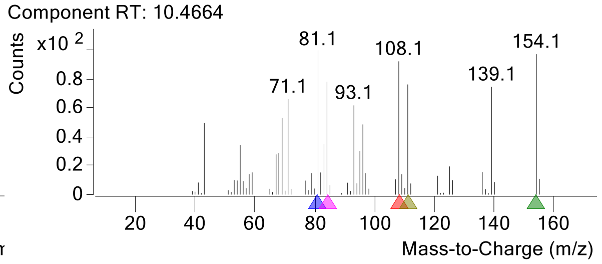

14. Eucalyptol

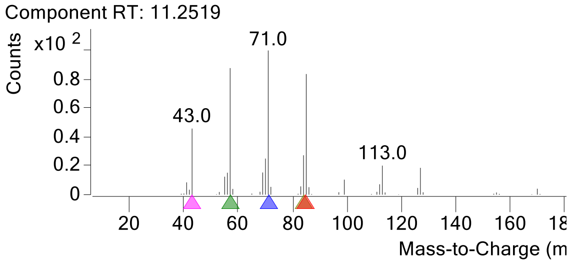

15. 4,5-Dimethylnonane

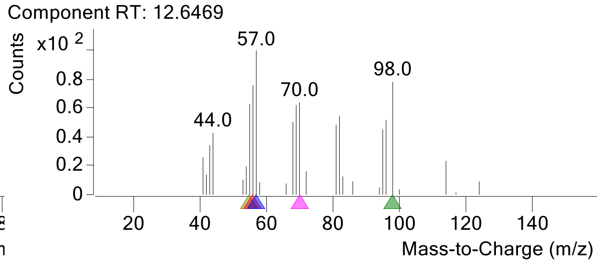

16. Nonanal

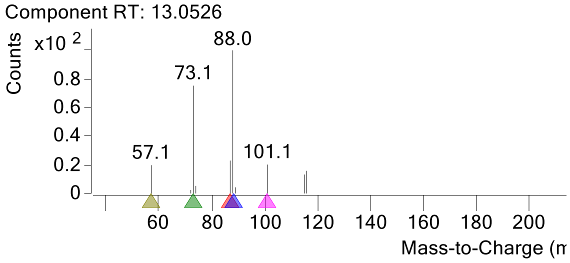

17. 2-Ethylhexanoic acid

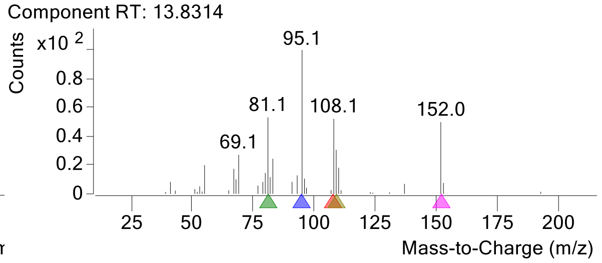

18. Camphor

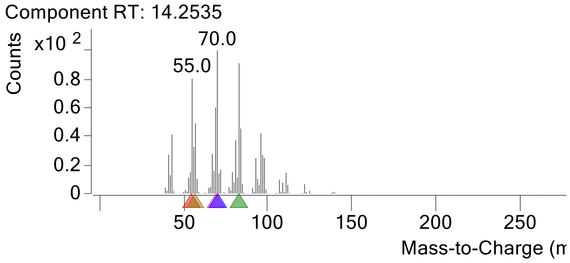

19. 2-Nonenal

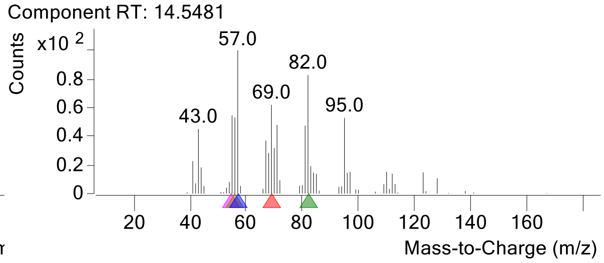

20. Octanoic acid

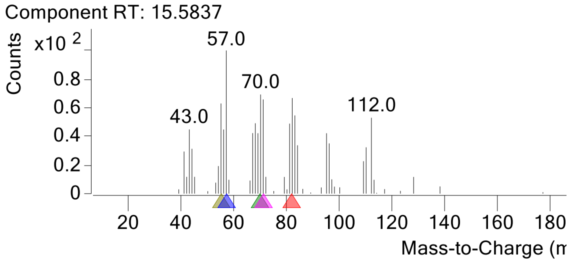

21. Decanal

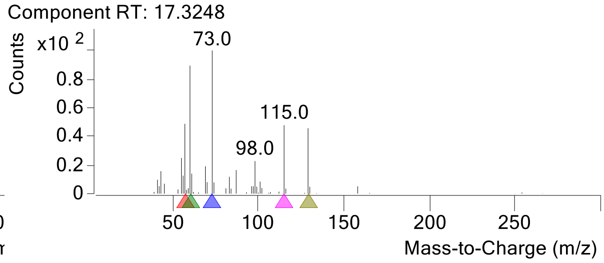

22. Nonanoic acid

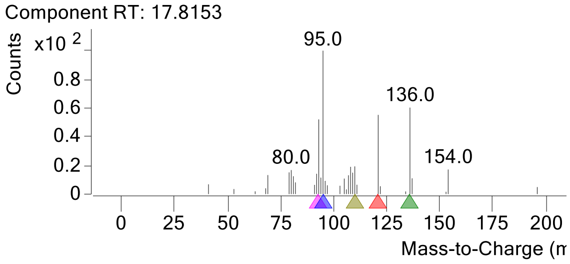

23. Bornyl acetate

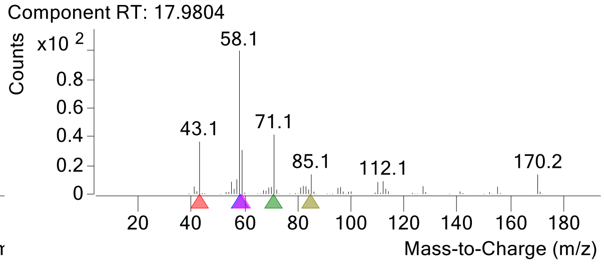

24. 2-Undecanone (IS)

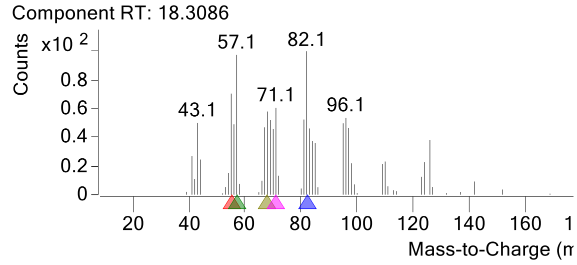

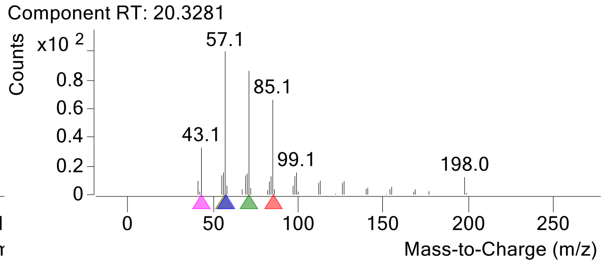

25. Undecanal

26. Tertadecane

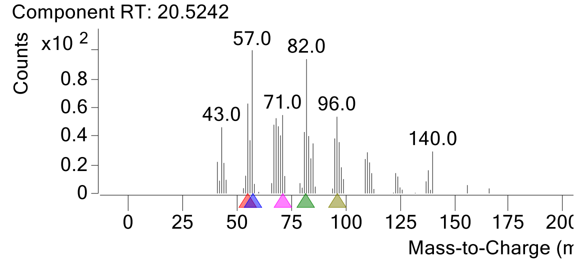

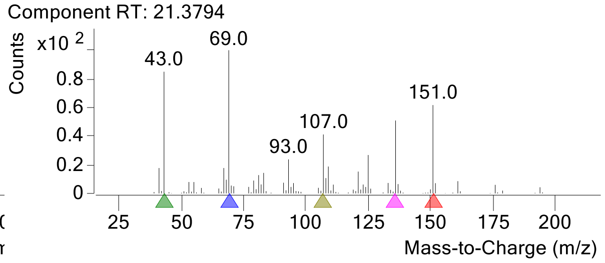

27. Dodecanal

28. trans-Geranylacetone

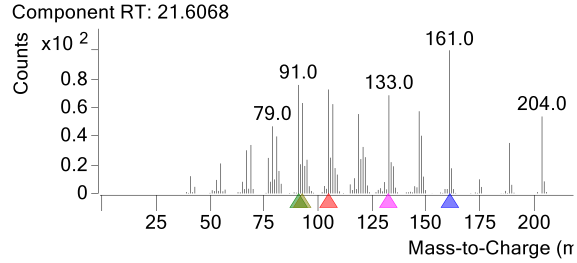

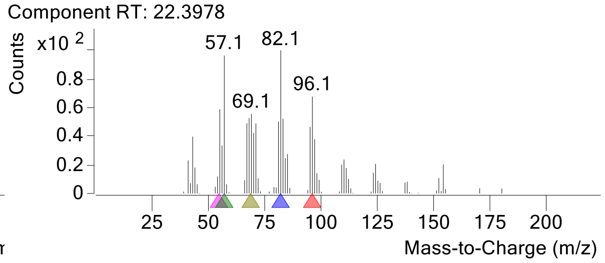

29. Alloaromadendrene

30. Tridecanal

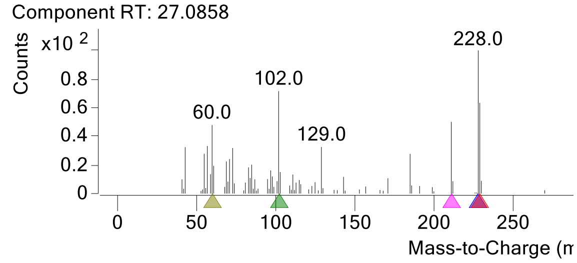

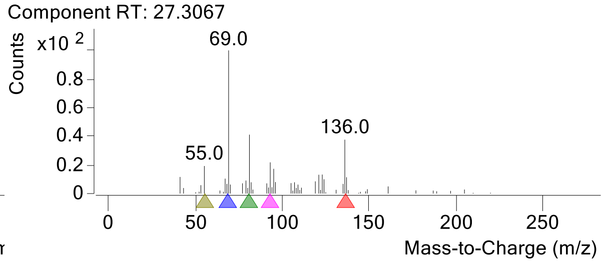

31. Isopropyl myristate

32. Farnesyl acetaldehyde

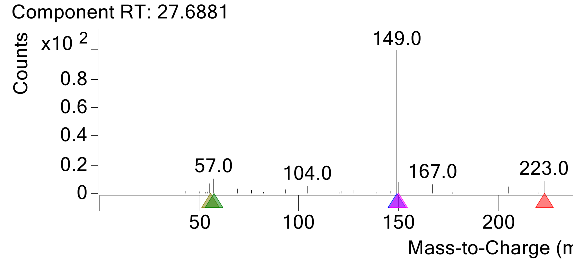

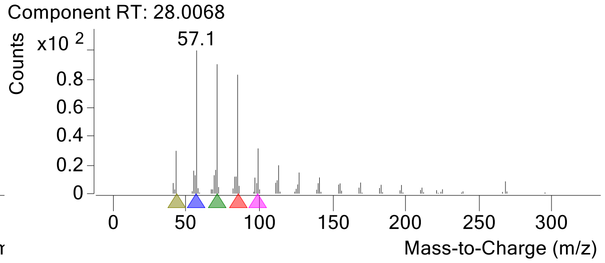

33. Phtalic acid, hept-4-yl isobutyl ester

34. Nonadecane

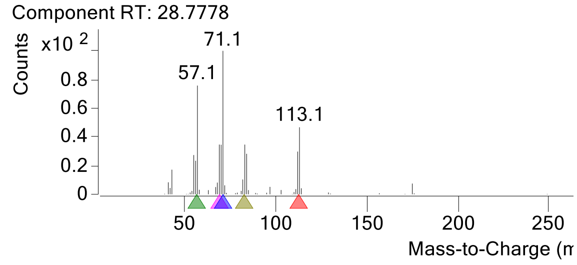

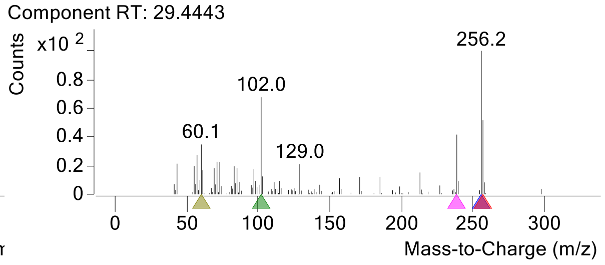

35. 2-Ethylhexyl octadecyl carbonate

36. Isopropyl palmitate

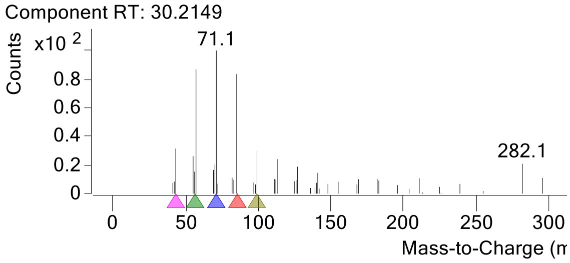

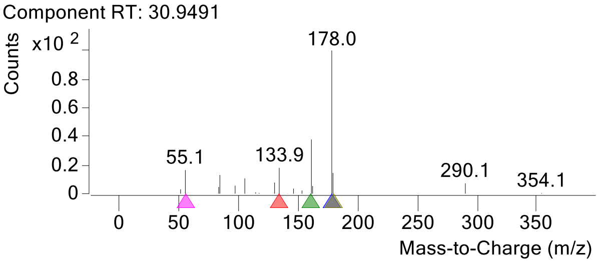

37. Heneicosane

38. 2-Ethylhexyl 4-methoxycinnamate
